# Evaluating effective measles vaccine coverage in the Malaysian population accounting for between-dose correlation and vaccine efficacy

**DOI:** 10.1186/s12889-023-17082-9

**Published:** 2023-11-28

**Authors:** Shurendar Selva Kumar, Anna-Maria Hartner, Arunah Chandran, Katy A. M. Gaythorpe, Xiang Li

**Affiliations:** 1 MRC Centre for Global Infectious Disease Analysis, Jameel Institute, School of Public Health, Imperial College London, London, W2 1NY UK; 2 Centre for Artificial Intelligence in Public Health Research, Robert Koch Institute, Wildau, Germany; 3 Lyon, France

**Keywords:** Vaccination Coverage, Measles Vaccine, Malaysia, Measles, Immunisation Programs, Vaccination, Dose correlation

## Abstract

**Background:**

Malaysia introduced the two dose measles-mumps-rubella (MMR) vaccine in 2004 as part of its measles elimination strategy. However, despite high historical coverage of MCV1 and MCV2, Malaysia continues to report high measles incidence. This study suggests a novel indicator for investigating population immunity against measles in the Malaysian population.

**Methods:**

We define effective vaccine coverage (EVC) of measles as the proportion of a population vaccinated with measles-containing vaccine (MCV) and effectively protected against measles infection. A quantitative evaluation of EVC throughout the life course of Malaysian birth cohorts was conducted accounting for both vaccine efficacy (VE) and between-dose correlation (BdC). Measles vaccination coverage was sourced from WHO-UNICEF estimates of Malaysia’s routine immunisation coverage and supplementary immunisation activities (SIAs). United Nations World population estimates and projections (UNWPP) provided birth cohort sizes stratified by age and year. A step wise joint Bernoulli distribution was used to proportionate the Malaysian population born between 1982, the first year of Malaysia’s measles vaccination programme, and 2021, into individuals who received zero dose, one dose and multiple doses of MCV. VE estimates by age and doses received are then adopted to derive EVC. A sensitivity analysis was conducted using 1000 random combinations of BdC and VE parameters.

**Results:**

This study suggests that no birth cohort in the Malaysian population has achieved > 95% population immunity (EVC) conferred through measles vaccination since the measles immunisation programme began in Malaysia.

**Conclusion:**

The persistence of measles in Malaysia is due to pockets of insufficient vaccination coverage against measles in the population. Monitoring BdC through immunisation surveillance systems may allow for the identification of susceptible subpopulations (primarily zero-dose MCV individuals) and increase the coverage of individuals who are vaccinated with multiple doses of MCV. This study provides a tool for assessment of national-level population immunity of measles conferred through vaccination and does not consider subnational heterogeneity or vaccine waning. This tool can be readily applied to other regions and vaccine-preventable diseases.

## Background

Measles is a highly infectious virus in the paramyxovirus family, transmitted through respiratory droplets or particle aerosols [1] with a basic reproduction number of 12 – 18 [2]. Despite significant progress in the global reduction of measles incidence and mortality since 2000, a joint report by the World Health Organization (WHO) and the United States Centers for Disease Control and Prevention (CDC) showed that the global incidence of measles rose to the highest level in 23 years in 2019, largely driven by large outbreaks in several countries [3]. Though the reasons for these outbreaks varied on a community level, low MCV1 coverage, decreases in vaccine confidence, and unidentified immunity gaps were identified as notable drivers [3].

Progress towards measles elimination targets as defined by the 2012 Global Vaccine Action Plan (GVAP) has regressed since the global resurgence of measles in 2017 [3], and despite the decline of measles cases by 80% in 2020 compared to the prior year, the COVID-19 pandemic also saw over 22 million children miss their first dose of measles vaccine alongside a decline in surveillance activities, leading a growing risk of further measles outbreaks as immunity gaps widen [4]. The theoretical threshold of population immunity required for the interruption of measles transmission is 92–94%, meaning that even in areas of high coverage, the spatial clustering of non-vaccinated individuals poses continued risks to achieving measles elimination, as observed in the outbreaks between 2017 and 2019 [3, 5, 6].

In 2009, updated WHO guidelines recommended MCV2 vaccination for all children, ensuring that individuals who missed the first dose or did not seroconvert after the first dose received a second opportunity at protection. A review of field effectiveness of measles vaccination found the median vaccine efficacy (VE) of first dose of MCV ranged from 84% (IQR 72.0%–95.0%) to 92.5% (IQR 84.8%–97.0%) depending on age; provision of a second dose of MCV increased VE point estimates to 94–100%, justifying the current coverage targets of 95% of both MCV1 and MCV2 and MCV2 introduction [7].

**Fig. 1:**
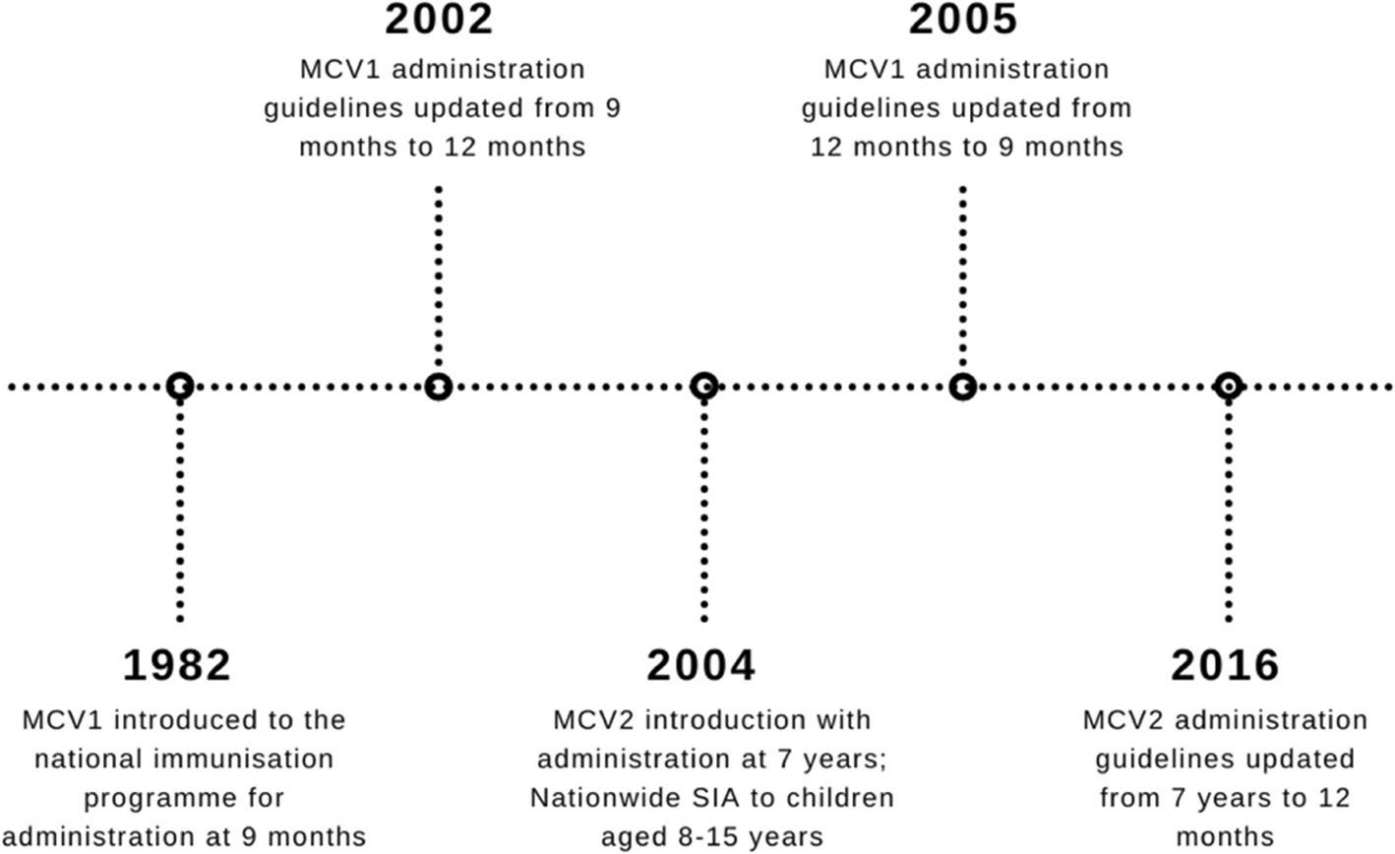
Changes to the Malaysian measles vaccination schedule and national measles immunisation campaigns

In Malaysia, the introduction of MCV1 in 1982 and MCV2 in 2004 led to a decline in measles incidence from 65.2 cases per million to 6.6 in 2013, in line with the expanded programme on immunisation and global measles elimination targets [8]. However, since 2013, increasing vaccine hesitancy and other implementation challenges have led to an increase in outbreaks, increasing incidence to 14.8 cases per million in 2020 [8, 9]. Currently, Malaysia administers MCV1 at 9 months of age and MCV2 at 12 months of age, a policy introduced in 2016 following a recommendation by the WHO [8]. Figure 1 summarises changes to the measles vaccination schedule and national measles immunisation campaigns since 1982.

Despite its public health benefits, the introduction of a second measles vaccine dose presents some programmatic challenges. In settings where there is low coverage of MCV1, implementing MCV2 routine or supplementary immunisation activities (SIAs) to increase the proportion of two-dose individuals may result in a smaller effect than reaching zero-dose MCV individuals [10]. Delayed 1st dose of MCV may also be incorrectly reported in administrative records as 2nd dose of MCV, leading to incorrect assumptions of overall population immunity [11]. Given VE, to effectively control outbreak risk, measles immunisation programmes aim to understand the relationship between zero-dose, MCV1, and MCV2 immunisation proportions in individuals [12]. To contribute to this understanding, this study suggests a novel concept—between dose correlation (BdC). It represents the correlation between whether an individual has been vaccinated previously and whether the individual will be vaccinated in the next round of immunisation activity.

For demonstration purposes, assume a cohort has been targeted in $i-1$ immunisation activities already, and is eligible for the ${i}^{th}$ immunisation activity. A perfect positive BdC means that the ${i}^{th}$ immunisation vaccine doses are primarily allocated to individuals already vaccinated previously. A random BdC assumes an equal likelihood of being vaccinated in the ${i}^{th}$ immunisation activity regardless of whether an individual has been vaccinated previously. A perfect negative correlation claims that individuals missing the previous $i-1$ immunisation activities are prioritised in the ${i}^{th}$ immunisation activity, resulting in fewer zero-dose individuals. With BdC, we are able to estimate the proportions of zero-dose, single-dose (or one-dose), and multiple-dose individuals for any birth cohort through time. In practice, surveillance systems are not designed to monitor BdC. Determining the relative proportions of single-, multiple- and zero-dose populations is therefore challenging. Vaccination programme managers tend to rely on aggregated subnational administrative and population data for coverage estimates [13], from which BdC is inferred.

These assumptions are critical for setting national vaccination targets and reducing the endemic transmission of measles. However, given recent outbreaks among older age groups, population immunity should be evaluated across the life course, accounting for both VE and BdC [14]. This study aims to determine the effective measles vaccine coverage (EVC) in Malaysian birth cohorts since 1982 using this approach with a particular focus on people under 18 years of age.

## Methodology

### Data sources

Measles routine immunisation coverage data from 1982 to 2021 in Malaysia was provided by WHO/UNICEF estimates of national immunisation coverage (WUENIC) [15], while WHO subnational campaign data was scaled to provide campaign coverage estimates at the national level. United Nations population estimates and projections (UNWPP) were used for cohort population sizes from the year 1982 until 2021 [16].

### Variable definitions

#### Between dose correlation (BdC)

Some papers on similar themes assume random correlation between vaccination activities [17, 18]. In this study, BdC represents the correlation between whether an individual has been vaccinated previously and whether the individual will be vaccinated in the next vaccination activity.

#### Vaccine efficacy (VE)

The probability that vaccination offers complete lifelong protection.

#### Effective measles vaccination coverage (EVC)

The proportion of a population/cohort effectively vaccinated against measles, i.e. an indicator of population immunity. It discounts individuals ineffectively vaccinated, i.e. those who are vaccinated but still susceptible to infection. Waning is not considered in this paper as it is out of scope. The evaluation of EVC is cohort-based and driven by vaccination coverage, BdC, and VE.

## Methods

The methodological basis for this evaluation was developed by the Vaccine Impact Modelling Consortium (VIMC) [19]. In the VIMC methodology, BdC is dependent on the number of doses an individual has been vaccinated with. In this paper, we simplify this by assuming the BdC is only related to whether an individual has been vaccinated previously. R programming software (version 4.0.2) was used for data management, analysis, and visualization.

Measles routine immunisation coverage estimates, i.e. MCV1 and MCV2, are sourced from the WHO routine immunisation data portal. Subnational supplementary immunisation activity (SIA) coverage estimates are scaled to the national level using vaccination doses divided by target population size. Vaccination doses through SIAs in the same year are aggregated assuming doses are allocated to different regions of a country. The target population size is defined through UNWPP estimates of population size by birth cohort and year.

To determine EVC of measles derived from vaccination in the Malaysian population, we first used a stepwise joint Bernoulli distribution to cluster individuals into those who have received zero, one, or multiple doses of measles vaccine, accounting for BdC. We then applied VE estimates to the clustered population to estimate EVC. Details of the approach are as follows.

For a birth cohort of interest, assume they (will) have benefited from $i \in \{1,...,K\}$ immunisation activities. We intend to cluster the cohort into zero-dose, single-dose (or one-dose), and multiple-dose populations through their life course. Denote random variables: ${V}_{i-1}$ as whether an individual is vaccinated in any of the first $i-1$ immunisation activities, ${M}_{i-1}$ as whether an individual is vaccinated with multiple doses through the first $i-1$ immunisation activities, and ${N}_{i}$ as whether an individual is vaccinated in the ${i}^{th}$ immunisation activity. Each of the three random variables follows a Bernoulli distribution. Assume the probability of each variable are ${v}_{i-1}$, ${m}_{i-1}$ and ${n}_{i}$, which represents the proportion of individuals vaccinated in the first $i-1$ immunisation activities, the proportion of multiple-dose population after the first $i-1$ immunisation activities, and vaccination coverage of the ${i}^{th}$ immunisation activity.

Further denote joint Bernoulli distributions $F({V}_{i-1},{N}_{i},{\theta }_{1})$ and $F({M}_{i-1},{N}_{i},{\theta }_{2})$, where ${\theta }_{1}$ and ${\theta }_{2}$ are corresponding BdC. In this study, we make a simplification on BdC by assuming ${\theta }_{1}={\theta }_{2}$. That means whether an individual is vaccinated in the ${i}^{th}$ immunisation activity is only dependent on if the individual has been vaccinated previously. We use these joint distributions to estimate vaccine coverage ${v}_{i}$ and multiple-dose proportion ${m}_{i}$. Zero- and Single-dose proportions are calculated as ${z}_{i}={1-v}_{i}$ and ${s}_{i}={v}_{i}-{m}_{i}$, respectively.

Set ${s}_{0}={m}_{0}={v}_{0} = 0$, we cluster a cohort into zero-, single- and multiple-dose categories for each $i$ step-wisely. Further taking into account vaccine efficacy (VE), effective vaccine coverage (EVC) can be calculated through $EV{C}_{i} ={s}_{i}*V{{E}^{s}}_{i}+{m}_{i}*V{{E}^{m}}_{i}$, where $V{E}^{s}$ and $V{E}^{m}$ represent single-dose and multiple-dose vaccine efficacy.

BdC data disaggregated by country is scarce, and in practice, ranges between -1 to 1 in the population due to varying vaccination strategies based on local context. As such, we conducted a sensitivity analysis by sampling 1,000 sets of BdC and VE values from distributions from available literature [7]. Table 1 and Table 2 depict the characteristics of input parameters for BdC and vaccine efficacy, respectively.

**Table 1:** Single dose and multi-dose vaccine efficacy according to age

Age	Single-dose vaccine efficacy (IQR)	Multiple-dose vaccine efficacy (IQR)	Distribution	Reference
0	0.84(0.72- 0.95)	0.94(0.88- 0.98)	Uniform	Uzicanin & Zimmerman [7]
1	0.92(0.85 -0.97)	0.94(0.88- 0.98)		
≥ 2	0.92(0.85–0.97)	0.94(0.88- 0.98)

**Table 2:** Characteristics of input parameters representing between dose correlation

Variable	Definition	Value(s)	Distribution	Assumption
cor(0,1)	Correlation between ‘dose zero’ and dose one	1	Fixed	Most commonly the administration of 1st dose of MCV
cor(1,2)	Correlation between first dose and second dose	-1 to 1	Uniform	Following administration of the 1st dose of MCV, subsequent doses are delivered as per the routine immunisation schedule or SIAs. Therefore, correlation parameter values are sampled randomly from a uniform distribution to reflect various real-life settings
cor(2,3)	Correlation between first two doses and third dose	-1 to 1	Uniform	
cor(3,4)	Correlation between first three doses and fourth dose	-1 to 1	Uniform	
cor(k-1,k), k > 5	Correlation between first k-1 doses and k^th^ dose	-1	Fixed	We assume that individuals are unlikely to receive subsequent doses should they have already received 4 doses (perfect negative BdC)

## Results

From 2005 to 2021, after the change to MCV1 administration from 12 to 9 months, our findings show that the proportion of children under 18 with multiple measles vaccine doses rises gradually from 45.9% (IQR 43.7—49.4%) to 56.8% (IQR 56.4—57.4%). Although Malaysia has had high MCV1 and MCV2 coverage (84–98%) since 2005, the proportion of multiple-dose population in the under-18 s is relatively low (< 60%). This is mainly due to the fact that MCV2 was introduced targeting age 7, and only shifted to age 1 from 2016. Specifically, cohorts born between 2010 and 2015 (age 6 to 11 by year 2021) are predominantly vaccinated with only one dose of MCV by year 2021. Concurrently, the number of children under 18 with a single dose decreases from 50.1% (IQR 43.0—54.4%) in 2005 to 42.2% (IQR 40.8—42.9%) in 2021 with the number of zero-dose children decreasing from 4.0% (IQR 1.9—7.6%) to 1.1% (IQR 0.6—1.9%) during the same period. The single dose population was only larger than the multi-dose population in the first year of MCV2 introduction in 2004, and Malaysia has consistently maintained > 95% coverage of children under 18 years of age receiving at least one dose of measles vaccination since the year 2005 in this subpopulation (Table 3). In the 17 years since the introduction of MCV2, national SIAs, and subnational SIAs, the single dose population has decreased by 15.77%.

**Table 3:** Proportion of children under 18 years old who have received one-dose and multiple-doses of MCV, and the estimated ‘zero-dose’ population by year

Year	Single-dose (%)	Multiple-dose (%)	Zero-dose (%)	EVC (%)
2005	50.1 (43.0—54.4)	45.9 (43.7—49.4)	4.0 (1.9—7.6)	86.7 (79.2—94.0)
2006	47.6 (42.5—51.0)	48.6 (47.0—51.3)	3.5 (2.0—6.2)	87.2 (80.3—94.0)
2007	45.9 (40.9—48.7)	50.7 (49.3—53.2)	3.4 (2.0—5.9)	87.4 (80.7—93.9)
2008	44.4 (39.9—46.9)	52.4 (51.2—54.7)	3.2 (2.0—5.5)	87.6 (81.1—93.9)
2009	43.4 (39.3—45.6)	53.6 (52.5—55.6)	3.0 (1.9—5.0)	87.8 (81.6—94.0)
2010	42.6 (39.0—44.5)	54.7 (53.7—56.4)	2.8 (1.8—4.5)	88.0 (81.8—94.1)
2011	41.6 (38.5—43.3)	55.8 (55.0—57.3)	2.6 (1.8—4.2)	88.2 (81.9—94.2)
2012	39.8 (36.3—42.6)	57.7 (55.6—59.8)	2.5 (1.7—3.9)	91.5 (86.3—95.7)
2013	40.8 (37.9—42.2)	56.7 (56.0—58.1)	2.5 (1.8—4.0)	91.4 (86.2—95.7)
2014	39.8 (37.9—41.8)	57.9 (56.5—58.8)	2.3 (1.6—3.7)	91.6 (86.4—95.9)
2015	39.6 (37.8—41.4)	58.1 (56.8—58.9)	2.3 (1.7—3.7)	91.6 (86.4—96.0)
2016	39.5 (38.6—40.7)	58.8 (57.8—59.9)	1.7 (1.3—2.6)	92.2 (87.1—96.1)
2017	39.1 (37.2—40.2)	59.8 (58.8—61.1)	1.2 (0.9—1.7)	92.8 (87.7—96.6)
2018	39.8 (37.6—40.9)	59.3 (58.5—60.6)	0.9 (0.5—1.8)	93.1 (87.9—97.0)
2019	40.3 (38.2 – 41.4)	58.9 (58.0—60.1)	0.9 (0.5—1.7)	93.1 (88.0—97.0)
2020	41.5 (40.3—42.0)	57.5 (57.2—58.0)	1.0 (0.6—1.7)	92.9 (87.9—96.8)
2021	42.2 (40.8—42.9)	56.8 (56.4 – 57.4)	1.1 (0.6—1.9)	92.9 (87.8—96.8)

**Fig. 2:**
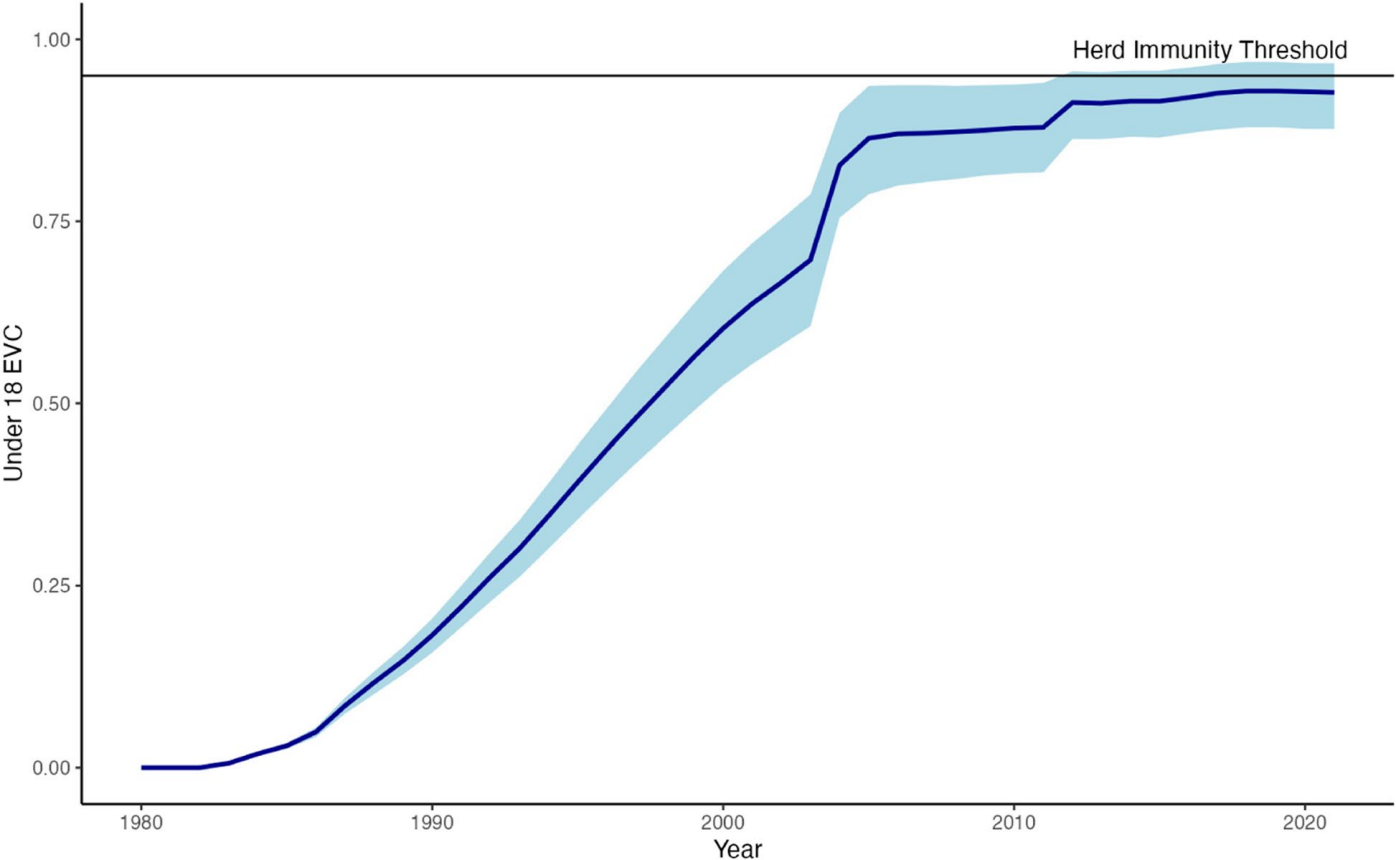
Effective measles vaccine coverage of the under 18 population by year. The blue ribbon represents the 95% credible interval for EVC estimates

There was a steady rise in under-18 s EVC from 1982 until 2003, followed by a sharp rise from 70.0% (60.6–79.1%) to 83.0% (75.9–90.1%) in 2004. Despite continued periods of increase in EVC following 2004, EVC never reaches the required 95% herd immunity target in children under 18 (Fig. 2).

**Fig. 3:**
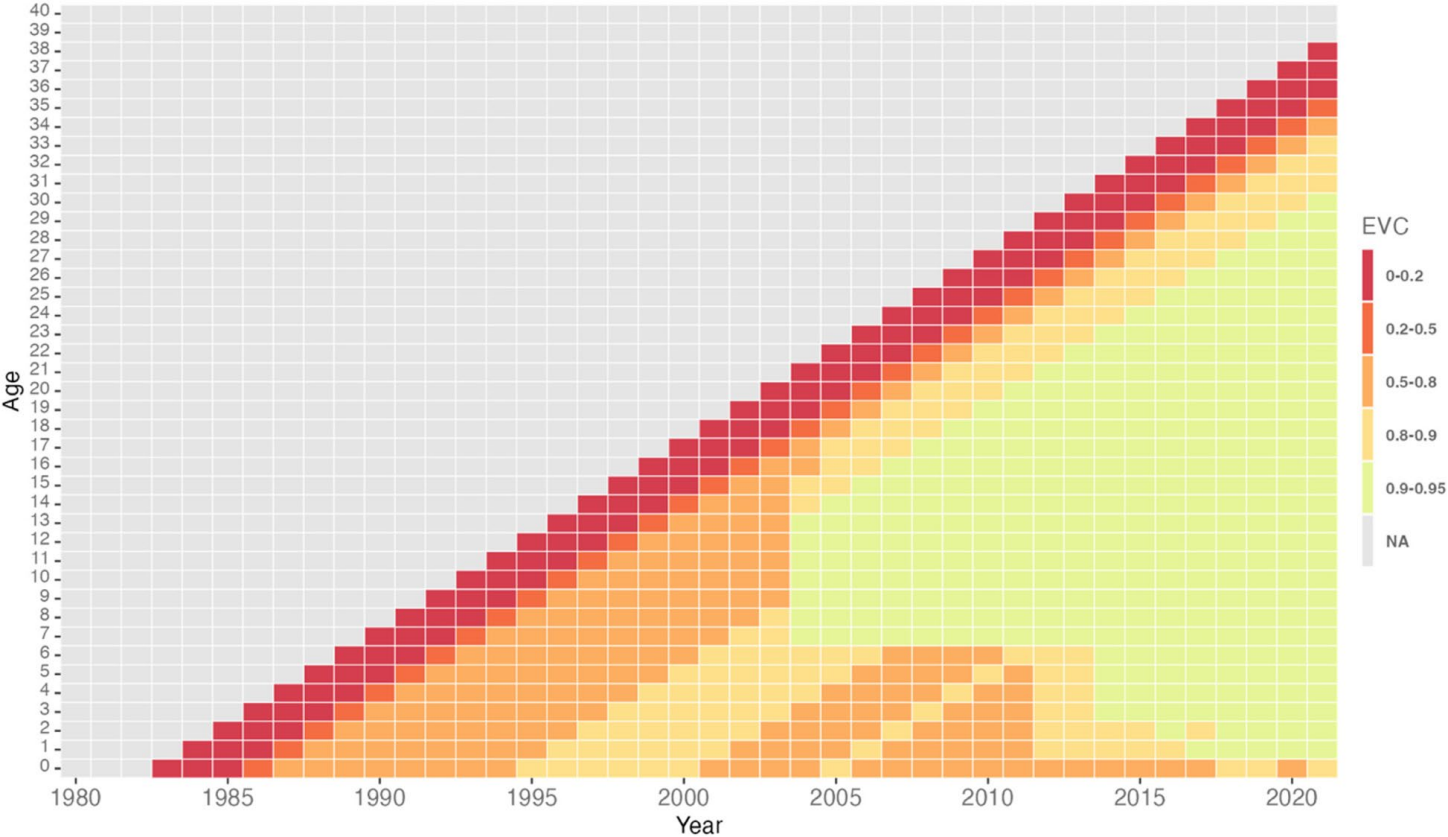
Relative levels of immunity of birth cohorts in the Malaysian population based on mean EVC values

The heatmap shown in Fig. 3 represents the relative levels of immunity of birth cohorts from 1982–2021 based on mean EVC values. Overall, the EVC of the Malaysian population represented here improves over time. The age at which mean EVC shifted to above 80% was between ages seven and fifteen prior to 2004; this has decreased to age one starting in 2011.

**Fig. 4:**
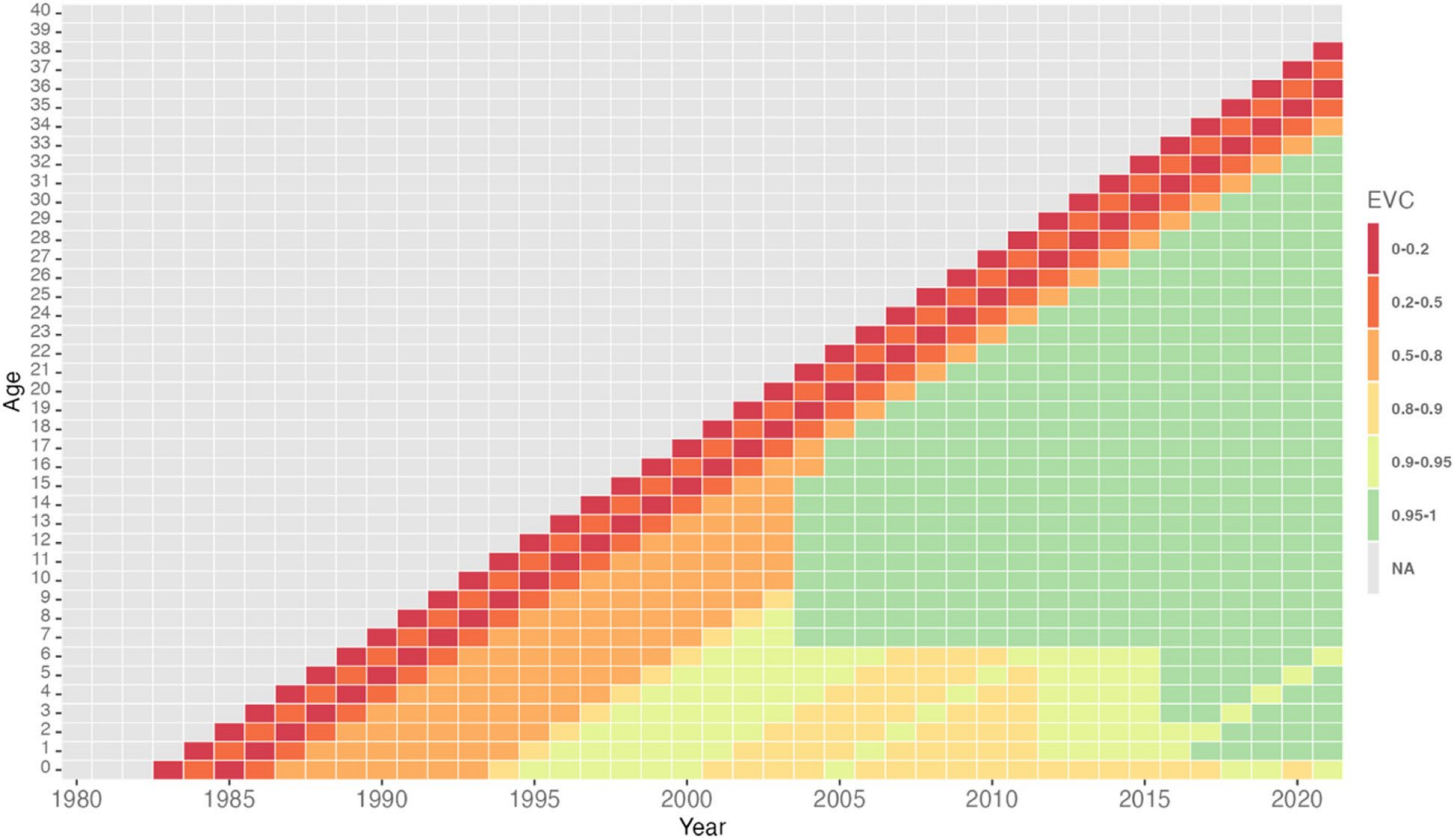
Relative levels of immunity of birth cohorts in the Malaysian population in 2.5% of the samples

Figure 4 shows the upper bound of 95% interquartile range of EVC in the Malaysian population, where some cohorts have crossed the 95% population immunity threshold beginning 2004, after the introduction of MCV2.

## Discussion

The Western Pacific Regional Plan of Action for Measles Elimination (2003) aims to ‘achieve and maintain 95% population immunity to measles in each birth cohort within each district of each country in the Region’ as one of its three main strategies towards measles elimination [20]. This is the first study that aims to do so by evaluating immunity conferred from measles vaccination in Malaysian birth cohorts throughout the life course. Our findings show that the Effective Vaccination Coverage (EVC) throughout the life course of Malaysian birth cohorts since 1982 is below the 95% immunity target threshold required to achieve measles elimination, contributing to the persistence of measles in Malaysia.

This study provides an assessment of national-level population immunity of measles, and does not consider subnational heterogeneity or vaccine waning. Additionally, there is no published literature as yet on national-level serological assessments of measles immunity. However, district and national laboratory serological assessments are available for comparison. One subnational study collected results of measles seroassays from September 2014 to January 2015 from government health clinics in a Malaysian district, r; here, individuals aged 15 to 24 years old showed a 74% positive titre (95% CI 70–78%) and children aged 6 to 9 years old showed 90% positive titre (95% CI 87–94%) [21]. A prior study conducted in the year 2008 using measles samples from Malaysian hospitals showed that 82.8% of individuals above the age of seven were seropositive for measles [22]. Malaysia has consistently reported more than 95% coverage of MCV1 and MCV2 in most years since 1982, yet the observations in this study and previous serological assessments show WUENIC estimates may not be the most accurate representation of measles population immunity in the country.

In 2013, updated guidelines for measles elimination were published by WPRO to recommend that countries ‘achieve and maintain > 95% vaccination coverage of two doses of MCV through routine immunisation, adding SIAs when required’ [23]. We find that the Malaysian population immunity conferred through measles vaccination increased significantly through the introduction of MCV2 and national immunisation campaigns. As noted in this study, a larger proportion of individuals receive MCV2 beginning in 2004; given that efficacy of measles vaccination improves with subsequent doses, EVC improves in these birth cohorts. Our findings corroborate this, showing that in 2.5% of samples, i.e. the best-case scenario, EVC crosses the 95% threshold. However, the study also finds that there is still a significant proportion of individuals in Malaysia who have not received any measles vaccination or only received MCV1.

These ‘best-case scenario’ findings, driven by higher vaccine efficacy, between dose coverage (BdC), proportion of individuals with MCV2, and a lower proportion of zero-dose individuals, represents the strategy required for immunisation programmes to meet the 95% threshold for measles control. Achieving these outcomes, however, requires immunisation programme capacity to monitor BdC. Since 1982, Malaysia has made significant strides towards the elimination of measles, implementing evidence-based vaccination policies that have resulted in a decreased burden. However, Malaysia is reliant on paper-based mechanisms for immunisation coverage reporting [24]. Paper-based systems depend on the aggregated facility or district-level data, and may be subject to delays or lags. This results in overreporting of estimates and increases the risk of unreadable, missing, or inconsistent vaccination coverage data. Electronic immunisation registries (EIR) circumvent these issues, especially ones that are interoperable with other electronic systems that handle patient records and civil registration systems for more accurate population and vaccine dose data [25, 26]. For Malaysia, a country aiming for measles elimination, the objective is to maintain high coverage and BdC through routine immunisation with periodic SIAs to cover immunity gaps. Conversely, in a country with a high burden of measles and low MCV1 coverage looking to achieve disease control, decorrelating measles vaccination doses provides a greater opportunity for zero-dose individuals to receive MCV1.

However, the implementation of a mechanism for monitoring BdC must consider contextual health system factors and feasibility. Developing health information management systems is costly and laborious, butalleviate user workload when compared to traditional paper-based systems [27, 28]. In this regard, there have been examples of innovative mixed paper and electronic immunisation registries that have shown promise in low-resource settings. Given the budgetary, infrastructural, and technical considerations at the national level in most digital health projects, it remains to be seen if this technology can be scalable [29].

There are several methodological strengths in this study. The use of probability theory to proportionate the population by the number of doses provides an opportunity to account for BdC in settings that do not have a mechanism for its monitoring. It also estimates the zero-dose population and gives insight into the effectiveness of SIAs in vaccinating susceptible populations. This is important as relying solely on vaccination coverage data may underestimate the proportion of susceptible individuals, as individuals with MCV1 may be subject to re-vaccination in these campaigns [30]. Future research should explore the age distribution of measles cases within the Malaysian population and correlate the findings of EVC by birth cohorts at the district level. This will better inform programme managers on the need for age-targeted immunisation campaigns as well as serological studies to evaluate population immunity against measles. This methodology provides flexibility in application, as it can be used for other multi-dose vaccination programmes.

Depending on the type of vaccine and efficacy data available, incorporating primary vaccination failure as a function of the methodology may need to be considered in subsequent research. This study uses the ‘all-or-nothing’ approach to define measles vaccine efficacy, and does not consider the waning of immunity conferred through vaccination as it does not play a significant role in measles transmission [31]. Future research should build upon the aforementioned aspects in order to provide more reliable estimates on the effectiveness of measles vaccination in a given population.

Uncertainty within our findings may stem from our use of UNWPP estimates of population demography and vaccination coverage estimates, given that the study methodology matched these estimates to immunisation data to allow for temporal analysis of population immunity. This may result in an overestimation or underestimation of the target population given that these are external sources of population estimates, and not in-country demographic statistics used by measles vaccine programme managers in Malaysia. Target population estimates influence the precision of vaccination coverage figures significantly, and increasingly so in settings with higher coverage levels [32]. Further, the temporal EVC changes seen within the population are based on vaccination delivery occurring as per the routine immunisation schedule. This may not necessarily reflect the situation in real life [33, 34]. Delays in vaccination administration may result in a child being part of the susceptible group for a longer period, shifting changes in EVC values in birth cohorts to higher age groups. Future research should explore the timeliness of measles vaccination to determine the proportion of children who receive measles immunisation at the recommended age.

Infants have protection from maternal antibodies against measles infection which wanes around 6 months from birth. The first dose of routine measles vaccination (MCV1) is usually given to infants at 9 months old. The methodology proposed in this paper does not account for maternal protection and the gap between its waning and the first dose of MCV vaccination, because this paper is primarily interested in vaccine induced immunity. Immunity induced by natural infection is also out of the scope in this paper. To be able to account for disease induced immunity, additional input of age specific measles cases and deaths are required. We consider this as a future research topic.

## Conclusion

While Malaysia has made considerable progress towards measles elimination, this study concludes that the current measles vaccination strategy in Malaysia has not conferred 95% population immunity in any of the Malaysian birth cohorts throughout the life course since 1982. Despite consistently maintaining > 95% MCV1 coverage of children under 18 years of age since the year 2005, Malaysia still reports measles cases. These findings highlight the need for the incorporation of BdC monitoring mechanisms into measles immunisation surveillance systems in Malaysia. Identifying immunity gaps and susceptible subpopulations will remain critical for immunisation programme strengthening.

## Data Availability

Measles vaccination coverage data was gathered from the World Health Organisation database. Available from: https://immunizationdata.who.int/pages/coverage/mcv.html. Malaysia population prospects was gathered from the Population Division, United Nations database. Available from: https://population.un.org/wpp/.

## Abbreviations

BdC: Between-dose Correlation
CDC: United States Centers for Disease Control and Prevention
EPI: Expanded Programme on Immunisation
EVC: Effective Vaccine Coverage
GAVI: Gavi, The Vaccine Alliance
JRF: Joint Reporting Form
MCV1: Measles-containing-vaccine first-dose
MCV2: Measles-containing-vaccine second-dose
MMR: Measles-Mumps-Rubella
SIA: Supplementary Immunisation Activities
UNICEF: United Nations Children’s Fund
UNWPP: United Nations Population Estimates and Projections
VPD: Vaccine-preventable Diseases
WHO: World Health Organization
WPRO: World Health Organization Regional Office for the Western Pacific
WUENIC: World Health Organization and United Nations Children’s Fund estimates of national immunization coverage
